# The Acceptability of Pre-Exposure Prophylaxis: Beliefs of Health-Care Professionals Working in Sexually Transmitted Infections Clinics and HIV Treatment Centers

**DOI:** 10.3389/fpubh.2018.00005

**Published:** 2018-02-09

**Authors:** Janneke P. Bil, Elske Hoornenborg, Maria Prins, Arjan Hogewoning, Fernando Dias Goncalves Lima, Henry J. C. de Vries, Udi Davidovich

**Affiliations:** ^1^Department of Infectious Diseases Research and Prevention, Public Health Service of Amsterdam, Amsterdam, Netherlands; ^2^Department of Infectious Diseases, STI Outpatient Clinic, Public Health Service of Amsterdam, Amsterdam, Netherlands; ^3^Department of Internal Medicine, Amsterdam Infection and Immunity Institute (AI&II), Academic Medical Center, University of Amsterdam, Amsterdam, Netherlands; ^4^Department of Dermatology, Academic Medical Center, University of Amsterdam, Amsterdam, Netherlands; ^5^Center for Infectious Disease Control, National Institute of Public Health and the Environment, Bilthoven, Netherlands

**Keywords:** HIV, pre-exposure prophylaxis, prevention, health personnel, implementation

## Abstract

**Background:**

Pre-exposure prophylaxis (PrEP) is highly effective for preventing HIV infections, but is not yet implemented in the Netherlands. As the attitudes of health-care professionals toward PrEP can influence future PrEP implementation, we studied PrEP knowledge and beliefs and their association with PrEP acceptability among professionals in clinics for sexually transmitted infection (STI professionals) and HIV treatment centers (HIV specialists). In addition, we examined preferred regimens, attitudes toward providing PrEP to key populations and to reimbursement of PrEP costs.

**Methods:**

An online questionnaire was distributed among 24 public health STI clinics and 27 HIV treatment centers nationwide in the Netherlands between January and August 2015. The acceptability of PrEP was measured on a 7-point Likert scale ranging from 1 = low to 7 = high acceptability. Univariable and multivariable linear regression analyses were used to explore associations between demographic characteristics, PrEP knowledge, beliefs about PrEP, and PrEP acceptability.

**Results:**

In total, 209 people (143 STI professionals and 66 HIV specialists) completed the questionnaire. The mean acceptability of PrEP implementation was 4.28 (SD 1.68) among STI professionals and 4.42 (SD 1.67) among HIV specialists. The mean score on self-perceived knowledge related to PrEP efficacy was 3.90 (SD 1.57) among STI professionals and 5.68 (SD 1.08) among HIV specialists (*p*-value of <0.001). Beliefs associated with lower PrEP acceptability among both groups were the fear that PrEP use will lead to a decrease in condom use and an increase in STI, the high costs of PrEP and ethical issues regarding prescribing antiretroviral medication to healthy individuals. No preference for a daily or an event-driven regimen was detected. Most participants deemed the following groups to be eligible for PrEP: men who have sex with men (MSM) who regularly get post-exposure prophylaxis, MSM who never used condoms with casual partners and MSM with an HIV-positive partner with a detectable viral load. Over half of the participants indicated that PrEP users should partly (54.1%) or fully (35.4%) pay the costs of PrEP.

**Conclusion:**

In 2015, PrEP acceptability was only moderate among Dutch STI professionals and HIV specialists, which is far from an optimal setting. Addressing barriers to PrEP acceptability in educational programs for various types of health-care professionals is needed to successfully implement PrEP in the Netherlands.

## Introduction

Pre-exposure prophylaxis (PrEP) entails offering a regime of lower-intensity antiretroviral therapy (ART) to HIV-negative individuals to reduce their risk of HIV infection. PrEP is offered in combination with other behavioral interventions such as condom use, counseling on sexual-risk behavior, frequent HIV testing, and linkage to other HIV prevention services (1–3). Several studies have demonstrated that both daily and event-driven PrEP regimens are highly effective (4–7), and the current guidelines of a number of international organizations include PrEP as a prevention strategy for key populations. In the developed world, HIV spreads most rapidly in men who have sex with men (MSM) (1, 8). A modeling study based on Dutch data estimated that 30% (range: 22–39%) of new HIV infections could have been averted if half of all MSM under 30 had received PrEP in combination with immediate ART for those who test positive (9). In addition, PrEP implementation was estimated to be cost-effective in the Netherlands over a 30-year time frame if PrEP was provided to the 10% most sexually active MSM (10). Despite these results and the July 2016 approval of Truvada (emtricitabine/tenofovir disoproxil) for PrEP in the European Union, PrEP is not yet routinely available or reimbursed in the Netherlands.

For the effective implementation of PrEP, it is important to understand health-care professionals’ level of knowledge and beliefs or concerns about PrEP, as they will play a critical role in its implementation. The acceptability of PrEP among health-care professionals at clinics for sexually transmitted infections (STIs) and HIV treatment centers is of particular importance as PrEP is most likely to be implemented at these facilities, due to their experience in offering prevention services to individuals at risk for HIV and experience in providing HIV treatment, respectively.

In this cross-sectional study among professionals working at STI clinics (STI professionals) and at HIV treatment centers (HIV specialists) in the Netherlands, we aimed to gain insight into the acceptability of PrEP, prescription setting, potential determinants of PrEP acceptability (such as knowledge and beliefs about PrEP), and preferred PrEP regimens. We examined attitudes toward providing PrEP to key populations and toward the reimbursement of PrEP costs. The resulting insights will guide future PrEP implementation strategies and help design PrEP educational programs for professionals.

## Materials and Methods

### Procedures and Recruitment

First, we conducted two focus group discussions with a total of 16 STI professionals from the Public Health Service of Amsterdam (three physicians, 12 nurses, and one doctor’s assistant) in October 2014. The central topics were PrEP acceptability and underlying PrEP beliefs. Each participant was asked about his/her opinion regarding PrEP implementation and was asked to shortly explain their most important argument(s) to support their opinion. All arguments were thereafter discussed within the group. The results of the focus group discussions were analyzed by three researchers from diverse disciplines: health sciences (JPB), medicine (EH), and psychology (UD). In the qualitative analyses, PrEP beliefs measured in the focus group discussions were categorized into major themes using thematic content analysis. Second, based on the focus group discussions, we developed two questionnaires. The first was aimed at STI professionals and included all PrEP beliefs measured in the focus group discussions. The second, aimed at HIV specialists, was condensed to encourage maximum response from this group. It included only the major themes derived from the qualitative analyses (Table S1 in Supplementary Material).

The questionnaire for STI professionals was distributed from January through May 2015 via email invitations to key contacts at all official public health STI clinics (*n* = 24) in the Netherlands. The contacts were asked to further distribute the questionnaire within their organization. The invitation and questionnaire for HIV specialists were emailed from June through August 2015 directly to physicians at all HIV treatment centers (*n* = 27) and to the nurse practitioners through their professional organization. In the email invitation for the questionnaire, participants were informed about PrEP, the aim of the study, and the sponsor of the study. One follow-up email was sent to increase the response rate. Participation was voluntary, and no financial incentive was offered. As the questionnaire was anonymous and did not request personal or sensitive data, approval by an ethics committee was not required, according to Dutch legislation.

### Measurements

#### Demographics, Characteristics, and PrEP Experience

Characteristics included age, gender, the type of professional (nurse, physician, or other, e.g., managers, doctors’ assistants, and administrative workers), the location of STI clinic (large urban area/outside of large urban area) or the type of hospital (academic or general), and the length of employment in STI clinic/hospital. HIV specialists were additionally asked for the number of HIV-positive patients at their clinic. Experience with PrEP was measured by asking whether participants had received questions about PrEP from clients/patients in the previous 6 months. HIV specialists were additionally asked whether they had ever prescribed PrEP.

#### PrEP Acceptability

Pre-exposure prophylaxis acceptability was measured via several items on a 7-point scale ranging from 1 “completely disagree” to 7 “completely agree.” The acceptability of PrEP implementation in the Netherlands was measured by one central item: “If PrEP is registered (i.e., granted market authorization), it should be implemented in the Netherlands as a new HIV intervention strategy.” In addition, among STI professionals, we also measured the acceptability of implementation at STI clinics by the item: “If PrEP is registered, it should be prescribed at STI clinics.” Among HIV specialists, we additionally measured acceptability toward implementation at HIV treatment centers and at general practitioners’ offices by the items: “If PrEP is registered, it should be prescribed by HIV specialists,” and “If PrEP is registered, it should be prescribed by general practitioners.” Moreover, we queried their willingness to prescribe through the item: “If PrEP is registered, I would be willing to prescribe PrEP.”

#### Self-Perceived Knowledge of PrEP

For all participants (except where indicated in parentheses), the following self-perceived items were measured on a 7-point Likert scale ranging from 1 “very poor” to 7 “very good”: knowledge of PrEP efficacy and the frequency/severity of side effects, the efficacy to inform patients about PrEP (only among STI professionals), the ability to identify key populations for PrEP use (only among HIV specialists), and the capability of deciding to prescribe PrEP (only among HIV specialists). Self-perceived knowledge about the frequency and severity of side effects was combined in one item in the analysis (self-perceived knowledge about side effects) as it was highly correlated (Spearman’s rho >0.9; *p* < 0.001).

#### Beliefs about PrEP

Based on the focus group discussions, general beliefs about PrEP were measured with 50 items among STI professionals and 15 items among HIV specialists (Table S1 in Supplementary Material). For analyses, items were combined if they measured the same belief and had acceptable correlation (two items: Spearman’s rho ≥0.6 and *p* < 0.05; more than two items: Cronbach’s alpha ≥0.7). As the data collection of general beliefs differed between STI professionals (where all beliefs from the focus group discussions were measured) and HIV specialists (where only the selected major themes from the focus group discussions were measured), these correlation analyses were done separately for STI professionals and HIV specialists, and no comparisons were performed between the groups regarding beliefs. This resulted in 16 general beliefs about PrEP in the questionnaire for STI professionals and eight in the questionnaire for HIV specialists. All general beliefs were measured on a 7-point scale ranging from 1 “completely disagree” to 7 “completely agree.”

Among STI professionals, we additionally measured one belief about PrEP efficacy (ranging from “PrEP can reduce the risk of HIV by 100%” to “Although PrEP reduces the risk of HIV, there is still a great risk of HIV transmission”): one belief about the frequency of side effects (side effects are rare/are sometimes reported/appear frequently/knowledge is still scarce/I don’t know) and one belief about the severity of side effects (side effects are often severe/are often mild or non-severe/knowledge is still scarce/I don’t know).

#### Daily versus Event-Driven PrEP Use

Beliefs about daily versus event-driven PrEP were measured with seven items in both questionnaires, using a 7-point scale ranging from 1 “completely disagree” to 7 “completely agree” (see Table S2 in Supplementary Material for more details). Given the good correlation between items (Cronbach’s alpha of 0.81 for STI professionals and 0.77 for HIV specialists), all seven items were combined.

#### Key Populations, Reimbursement of PrEP Costs, and Serodiscordant Couples

Questions were asked about the perceived PrEP eligibility of key groups (MSM, heterosexuals, and commercial sex workers) within different behavioral scenarios (e.g., condom use, repeatedly having an STI, having an HIV-positive partner, and repeatedly using post-exposure prophylaxis). For each scenario, participants were asked to assess eligibility by choosing one of the following options: not eligible/possibly eligible/eligible.

Participants were also asked who should pay for PrEP (PrEP user/health insurance/government/partially PrEP users and partially health insurance or government/neutral).

HIV specialists were asked what intervention they prefer to prevent HIV transmission in serodiscordant couples (only ART for the HIV-positive partner/only PrEP for the HIV-negative partner/ART for the HIV-positive partner and PrEP for the HIV-negative partner as a transition phase until the HIV-positive partner has an undetectable viral load/only counseling).

### Statistical Analyses

We described demographic characteristics, PrEP experience and knowledge, general beliefs about PrEP and about event-driven versus daily use, key populations, reimbursement of costs, and preferred interventions for serodiscordant couples. We compared distributions of acceptability and knowledge by the type of health-care provider (nurse/physician/other profession) and between STI professionals and HIV specialists, using Student’s *t*-tests and one-way ANOVA for normally distributed continuous variables and Mann–Whitney *U*-tests and Kruskal–Wallis tests for not-normally distributed continuous variables. Because of the differences in how the general beliefs were constructed among STI professionals and HIV specialists, a direct comparison between the general beliefs between STI clinic professionals and HIV specialists was not possible.

We performed univariable and multivariable linear regression analyses to explore associations between demographic characteristics, PrEP knowledge, PrEP beliefs, and the acceptability of PrEP implementation in the Netherlands. A multivariable model was built using backward techniques. First, variables on PrEP knowledge and beliefs with a *p*-value of <0.10 in univariable analyses were entered into the model. Second, also demographic determinants with a *p* of <0.10 in univariable analyses were entered into the model. A *p*-value of ≤0.05 was considered to be statistically significant. Interactions between the variables in the final model were checked. As the data collection differed between STI professionals and HIV specialists, analyses were done separately for STI professionals and HIV specialists. Analyses were performed using STATA Intercooled 13.1 (STATA Corporation, College Station, TX, USA).

## Results

### Demographic Characteristics and PrEP Experience

**Figure 1:**
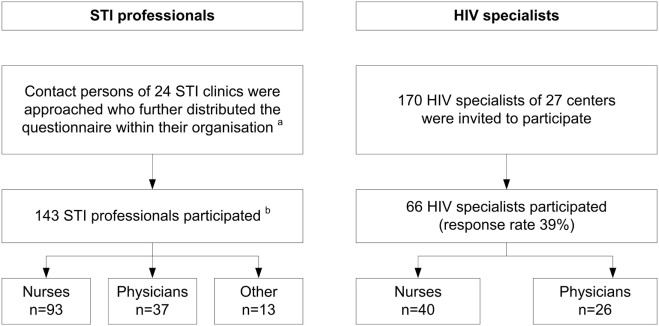
Flowchart of recruitment procedures of STI professionals working at STI clinics of the Public Health Service and HIV specialists working at HIV treatment centers, the Netherlands, 2015. (a) Total number of persons invited by contact persons is unknown. (b) Response rate was 54% (104/191) among nine clinics that provided data on the number of questionnaires distributed. STI, sexually transmitted infection.

All of the 24 STI clinics we contacted agreed to participate in the study (Figure 1). In the nine STI clinics that provided data on the number of questionnaires distributed, the response rate was 54.4% (104/191). In total, 143 STI professionals completed the questionnaire (Table 1): 93 nurses, 37 physicians, and 13 other professionals. A majority of respondents (113/143, 79.0%) were female; about half worked in an STI clinic in a large urban area (65/143, 45.5%) and most (95/143, 66.4%) had worked for more than 4 years in an STI clinic. About half (69/143, 48.3%) had received questions about PrEP from clients in the preceding 6 months but only 4.3% (3/69) were queried at least once a week.

**Table 1:** Demographic characteristics and PrEP experience of 209 health-care professionals the Netherlands (2015).

	STI professionals								HIV specialists					
	Total (*N* = 143)		Nurse (*n* = 93)		Physician (*n* = 37)		Other (*n* = 13)		Total (*N* = 66)		Nurse (*n* = 40)		Physician (*n* = 26)	
	*n*	%	*n*	%	*n*	%	*n*	%	*n*	%	*n*	%	*n*	%
**Age**														
<40 years	57	39.9%	29	31.2%	20	54.1%	8	61.5%	11	16.7%	7	17.5%	4	15.4%
40–49 years	41	28.7%	33	35.5%	6	16.2%	2	15.4%	27	40.9%	16	40.0%	11	42.3%
50–59 years	34	23.8%	25	26.9%	6	16.2%	3	23.1%	19	28.8%	14	35.0%	5	19.2%
≥60 years	11	7.7%	6	6.5%	5	13.5%	0	0.0%	9	13.6%	3	7.5%	6	23.1%

**Gender**														
Male	30	21.0%	13	14.0%	14	37.8%	3	23.1%	27	40.9%	12	30.0%	15	57.7%
Female	113	79.0%	80	86.0%	23	62.2%	10	76.9%	39	59.1%	28	70.0%	11	42.3%

**Location of STI clinic**														
Large urban area (Amsterdam, The Hague, Rotterdam, Utrecht)	65	45.5%	42	45.2%	15	40.5%	8	61.5%	NA					
Outside large urban area	75	52.5%	50	53.8%	20	54.1%	5	38.5%	NA					
Missing data	3	2.1%	1	1.1%	2	5.4%	0	0.0%						

**Type of hospital**														
Academic	NA								32	48.5%	18	45.0%	14	53.8%
General	NA								34	51.5%	22	55.0%	12	46.2%

**Length of employment in STI clinic/hospital**														
0–4 years	48	33.6%	24	25.8%	18	48.7%	6	46.2%	10	15.2%	7	17.5%	3	11.5%
5–9 years	45	31.5%	33	35.5%	11	29.7%	1	7.7%	18	27.3%	10	25.0%	8	30.8%
10–14 years	31	21.7%	26	28.0%	4	10.8%	1	7.7%	21	31.8%	17	42.5%	4	15.4%
≥15 years	19	13.3%	10	10.8%	4	10.8%	5	38.5%	17	25.8%	6	15.0%	11	42.3%

**Number of HIV-positive patients in care**														
≥250	NA								25	37.9%	10	25.0%	15	57.7%
>250	NA								41	62.1%	30	75.0%	11	42.3%

**Questions about PrEP from clients in the previous 6 months**														
No questions	65	45.5%	40	43.0%	22	59.5%	3	23.1%	11	16.7%	5	12.5%	6	23.0%
Very rarely (one to two in the preceding 6 months)	51	35.7%	41	44.1%	7	18.9%	3	23.1%	28	42.4%	16	40.0%	12	46.2%
Sometimes (one to two per month)	15	10.5%	10	10.8%	3	8.1%	2	15.4%	24	36.4%	16	40.0%	8	30.8%
Regularly (at least once a week)	3	2.1%	2	2.2%	1	2.7%	0	0.0%	3	4.5%	3	7.5%	0	0.0%
NA (no contact with clients)	9	6.3%	0	0.0%	4	10.8%	5	38.5%	0	0.0%	0	0.0%	0	0.0%

**Ever prescribed PrEP**														
No	NA								56	84.9%	31	77.5%	25	96.2%
Yes	NA								5	7.6%	4	10.0%	1	3.9%
NA	NA								5	7.6%	5	12.5%	0	0.0%

*NA, not applicable (item not included in the questionnaire for the indicated practice setting); PrEP, pre-exposure prophylaxis; STI, sexually transmitted infection*.

Of 170 HIV specialists invited to participate, 66 (38.8%) completed the questionnaire, including 40 nurses and 26 physicians (Table 1). Most HIV specialists were female (39/66, 59.1%); half (34/66, 51.5%) worked in an academic hospital and 84.8% (56/66) had more than 4 years of experience. Of these specialists, 83.3% (55/66) had received patient questions about PrEP in the preceding 6 months and 7.6% (5/66) had ever prescribed PrEP.

### PrEP Acceptability

Among STI professionals and HIV specialists, the mean score on the acceptability of PrEP implementation in the Netherlands was 4.28 (SD 1.68) and 4.42 (SD 1.67), respectively (Table 2). The mean acceptability score of PrEP implementation at STI clinics was 4.16 (SD 1.86) among STI professionals and 4.48 (SD 1.83) among HIV specialists. Among the latter, the mean acceptability score for implementation at HIV treatment centers was 3.91 (SD 1.97) and 2.45 (SD 1.82) for implementation at general practitioners’ offices. Their mean score for the willingness to prescribe PrEP was 4.39 (SD 1.89).

**Table 2:** Knowledge and beliefs about PrEP among 209 health-care professionals, the Netherlands (2015).

	STI professionals									HIV specialists							STI professionals versus HIV specialists
	Total (*N* = 143)		Nurse (*n* = 93)		Physician (*n* = 37)		Other (*n* = 13)		STI nurse versus STI physician versus STI other	Total (*N* = 66)		Nurse (*n* = 40)		Physician (*n* = 26)		HIV nurse versus HIV physician	
	Mean	SD	Mean	SD	Mean	SD	Mean	SD		Mean	SD	Mean	SD	Mean	SD		
**PrEP acceptability (7-point scale: 1 “completely disagree” to 7 “completely agree”)**																	
If PrEP is registered, it should be implemented in the Netherlands as a new HIV intervention strategy	4.28	1.68	4.20	1.56	4.00	1.84	5.62	1.56	*F*(2,140) = 4.98, *p* = 0.008	4.42	1.67	4.73	1.47	3.96	1.89	*t*(64) = −1.84, *p* = 0.070	*t*(207) = −0.58, *p* = 0.563

If PrEP is registered, it should be prescribed at STI clinics	4.16	1.86	4.01	1.75	4.14	2.07	5.31	1.70	*F*(2,140) = 2.86, *p* = 0.061	4.48	1.83	4.53	1.85	4.42	1.84	*t*(64) = −0.22, *p* = 0.827	*z* = −1.22, *p* = 0.224

If PrEP is registered, it should be prescribed by HIV specialists	NA									3.91	1.97	4.03	2.06	3.73	1.87	*z* = −0.66, *p* = 0.510	

If PrEP is registered, it should be prescribed by general practitioners	NA									2.45	1.82	2.65	1.94	2.15	1.62	*z* = −0.97, *p* = 0.333	

If PrEP is registered, I would be willing to prescribe PrEP	NA									4.39	1.89	4.55	1.72	4.15	2.13	*z* = −0.69, *p* = 0.489	

**Self-perceived knowledge of PrEP (7-point scale: 1 “very poor” to 7 “very good”)**																	
Self-perceived knowledge of PrEP efficacy	3.90	1.57	3.67	1.49	4.35	1.64	4.23	1.74	*F*(2,140) = 2.91, *p* = 0.058	5.68	1.08	5.48	1.11	6.00	0.98	*z* = 1.89, *p* =0.059	*z* = −7.46, *p* <0.001

Self-perceived knowledge of PrEP side effects	2.84	1.39	2.68	1.26	3.36	1.53	2.46	1.63	*H*(2) = 6.21, *p* = 0.045	5.61	1.34	5.28	1.36	6.12	1.15	*z* = 2.80, *p* =0.005	*z* = −9.60, *p* <0.001

Self-perceived efficacy to inform clients about PrEP	2.89	1.44	2.69	1.22	3.41	1.72	2.85	1.77	*H*(2) = 4.35, *p* = 0.114	NA							

Self-perceived ability to identify key populations for PrEP	NA									5.02	1.33	4.95	1.38	5.12	1.28	*t*(64) = 0.49, *p* = 0.625	

Self-perceived capability of deciding to prescribe PrEP	NA									4.61	1.40	4.33	1.37	5.04	1.37	*t*(64) = 2.07, *p* = 0.042	

**General beliefs about PrEP (7-point scale: 1 “completely disagree” to 7 “completely agree”)**																	
It is unclear who has to pay for PrEP	5.52	1.51	5.58	1.46	5.57	1.66	5.00	1.41	*H*(2) = 2.41, *p* = 0.300	NA							

Taking PrEP is better than getting HIV	5.27	1.49	5.09	1.59	5.51	1.26	5.85	1.14	*H*(2) = 3.53, *p* = 0.171	NA							

Adherence to PrEP will be insufficient	5.25	1.41	5.21	1.44	5.46	1.40	4.92	1.20	*H*(2) = 2.55, *p* = 0.280	NA							

The role of the pharmaceutical companies in regard to PrEP is unclear	4.97	1.43	5.06	1.47	4.65	1.25	5.23	1.59	*H*(2) = 4.15, *p* = 0.126	NA							

The use of PrEP will lead to a decrease in condom use and an increase in STI	4.87	1.21	5.07	1.08	4.66	1.34	4.02	1.32	*F*(2,140) = 5.42, *p* = 0.005	5.13	1.34	5.10	1.37	5.17	1.32	*z* = 0.27, *p* = 0.785	

PrEP is cheaper than lifelong HIV treatment	4.86	1.49	4.72	1.48	5.05	1.54	5.31	1.38	*F*(2,140) = 1.31, *p* = 0.274								

PrEP is an effective intervention to prevent HIV	4.74	1.36	4.68	1.33	4.65	1.52	5.46	0.97	*H*(2) = 3.67, *p* = 0.160	3.88	0.89	3.87	0.91	3.88	0.89	*t*(64) = 0.06, *p* = 0.954	

PrEP prescription should be part of routine care at STI clinics	4.67	1.66	4.74	1.62	4.20	1.76	5.54	1.38	*H*(2) = 6.99, *p* = 0.030	NA							

There is not enough knowledge yet about PrEP	4.67	1.23	4.84	1.18	4.48	1.31	3.98	1.09	*F*(2,140) = 3.45, *p* = 0.034	NA							

Costs of PrEP are a problem	4.66	1.16	4.65	1.09	4.87	1.24	4.12	1.29	*F*(2,140), *p* = 0.134	NA							

I would worry that some people have to use PrEP lifelong	4.56	1.76	4.74	1.71	4.43	1.68	3.62	2.10	*H*(2) = 4.08, *p* = 0.130	NA							

It is unethical to prescribe ART to healthy individuals	4.31	1.49	4.44	1.48	4.24	1.42	3.62	1.69	*F*(2,140) = 1.79, *p* = 0.170	4.64	1.72	4.85	1.41	4.31	2.09	*z* = −0.91, *p* = 0.364	

PrEP is a good addition to prevention strategies	4.27	1.07	4.28	1.01	4.04	1.15	4.80	1.14	*F*(2,140) = 2.53, *p* = 0.083	4.23	1.59	4.38	1.39	4.00	1.85	*z* = −0.68, *p* = 0.495	

The costs of PrEP will not outweigh the number of HIV infection prevented	3.97	1.42	4.18	1.33	3.81	1.54	2.85	1.14	*F* (2,140) = 5.69, *p* = 0.004	NA							

The STI clinic is not the right place for PrEP prescription	3.72	1.12	3.75	1.12	3.72	1.16	3.57	1.15	*F*(2,140) = 0.14, *p* = 0.869	NA							

The daily use of prevention strategies has already been tested	3.35	1.58	3.51	1.51	2.78	1.62	3.85	1.72	*F*(2,140) = 3.57, *p* = 0.031	NA							

I’m worried about the long-term side effects of PrEP	NA									4.89	1.51	5.05	1.41	4.65	1.65	*z* = −0.90, *p* = 0.366	

PrEP will lead to an increase in HIV resistance	NA									4.24	1.49	4.28	1.41	4.19	1.63	*t*(64) = 0.22, *p* = 0.828	

Non-biomedical HIV interventions (i.e., behavioral) are better than PrEP	NA									3.97	1.40	4.03	1.27	3.88	1.61	*t*(64) = −0.39, *p* = 0.694	

I’m worried about the short-term side effects of PrEP	NA									3.12	1.53	3.43	1.63	2.65	1.26	*t*(64) = −2.04, *p* = 0.045

*ART, antiretroviral therapy; NA, not applicable (item not included in the questionnaire for the indicated practice setting); PrEP, pre-exposure prophylaxis; SD, standard deviation; STI, sexually transmitted infection*.

### Self-Perceived Knowledge of PrEP

Among STI professionals, the mean score on self-perceived knowledge was 3.90 (SD 1.57) for PrEP efficacy and 2.84 (SD 1.39) for PrEP side effects; the mean score on self-perceived efficacy to inform clients about PrEP was 2.89 (SD 1.44) (Table 2). The mean score on self-perceived knowledge among HIV specialists was 5.68 (SD 1.08) for PrEP efficacy and 5.61 (SD 1.34) for side effects; it was 5.02 (SD 1.33) for self-perceived efficacy to identify key groups and 4.61 (SD 1.40) for the decision to prescribe PrEP. Self-perceived knowledge of efficacy (*z* = −7.46, *p* < 0.001) and side effects (*z* = −9.60, *p* < 0.001) was significantly higher among HIV specialists compared to that among STI professionals.

### Beliefs about PrEP

Among STI professionals, the three general beliefs with the strongest agreement scores were (Table 2) “it is unclear who will have to pay for PrEP” (mean 5.52, SD 1.51), “taking PrEP is better than getting HIV” (mean 5.27, SD 1.49), and “adherence to PrEP will be insufficient” (mean 5.25, SD 1.41). Among HIV specialists, the three general beliefs with the highest agreement scores were “the use of PrEP will lead to a decrease in condom use and an increase in STI” (mean 5.13, SD 1.34), “I’m worried about long-term side effects of PrEP” (mean 4.89, SD 1.51), and “it is unethical to prescribe ART to healthy individuals” (mean 4.64, SD 1.72).

### Determinants of PrEP Acceptability

In the final multivariable analyses among STI professionals (Table 3), the following beliefs were associated with a higher acceptability of PrEP implementation in the Netherlands: “taking PrEP is better than getting HIV” (β = 0.15, *p* = 0.020), “PrEP is an effective intervention to prevent HIV” (β = 0.45, *p* < 0.001), and “PrEP prescription should be part of routine care at STI clinics” (β = 0.17, *p* = = 0.013). Beliefs associated with a lower PrEP acceptability among STI professionals were “the use of PrEP will lead to a decrease in condom use and increase in STI” (β = −0.21, *p* = 0.034) and “the costs of PrEP are a problem” (β = −0.28, *p* = 0.019).

**Table 3:** Determinants of acceptability of PrEP implementation in the Netherlands among 143 STI professionals in the Netherlands (2015).

	Univariable analyses			Multivariable analyses		
	β	(95% CI)	*p*-value	β	(95% CI)	*p*-value
**Age**						
<40 years	Ref.		0.101			
40–49 years	0.20	(−0.47: 0.88)				
50–59 years	0.38	(−0.33: 1.09)				
≥60 years	−0.03	(−2.11: 0.05)				

**Gender**						
Male	Ref.		0.031			
Female	−0.74	(−1.42: −0.07)				

**Profession**						
Nurse	Ref.		0.008			
Physician	−0.20	(−0.83: 0.42)				
Other	1.41	(0.45: 2.37)				

**Location of STI clinic**						
Large urban area (Amsterdam, The Hague, Rotterdam, Utrecht)	Ref.		0.027			
Outside large urban area	0.63	(0.07: 1.19)				

**Length of employment within STI clinic**						
0–4 years	Ref.		0.069			
5–9 years	0.14	(−0.54: 0.82)				
10–14 years	−0.07	(−0.83: 0.68)				
≥15 years	1.11	(0.22: 1.99)				

**Questions about PrEP from clients in the previous 6 months**						
No	Ref.		0.201			
Yes	0.36	(−0.19: 0.92)				

**Self-perceived knowledge of PrEP (7-point scale: 1 “very poor” to 7 “very good”)**						
Self-perceived knowledge of PrEP efficacy	0.31	(0.14: 0.48)	<0.001			
Self-perceived knowledge of PrEP side effects	0.31	(0.11: 0.50)	0.002			
Self-perceived efficacy to inform clients about PrEP	0.31	(0.13: 0.50)	0.001			

**Beliefs about the efficacy of PrEP**						
PrEP can reduce the risk of HIV by 100%	Ref.		0.004			
PrEP can significantly reduce the risk of HIV	−1.55	(−3.83: 0.73)				
Although PrEP reduces the risk of HIV, there is still a great risk of HIV transmission	−2.83	(−5.44: −0.22)				
I don’t know	−2.93	(−5.35: −0.51)				

**Beliefs about the frequency of PrEP side effects**						
Side effects are rare	Ref.		0.006			
Side effects are sometimes reported	−1.09	(−2.59: 0.41)				
Side effects appear frequently	−1.98	(−3.57: −0.40)				
Knowledge about side effects is still scarce	−1.30	(−2.92: 0.32)				
I don’t know	−2.04	(−3.55: −0.53)				

**Beliefs about the severity of PrEP side effects**						
Side effects are often severe	Ref.		0.084			
Side effects are often mild/non-severe	1.13	(−2.26: 2.53)				
Knowledge about the severity of side effects is still scarce	0.70	(−0.83: 2.23)				
I don’t know	0.42	(−0.10: 1.84)				

**General beliefs about PrEP (7-point scale: 1 “completely disagree” to 7 “completely agree”)**						
It is unclear who has to pay for PrEP	−0.11	(−0.30: 0.07)	0.222			
Taking PrEP is better than getting HIV	0.59	(0.43: 0.75)	<0.001	0.15	(0.02: 0.27)	0.020
Adherence to PrEP will be insufficient	−0.36	(−0.55: −0.17)	<0.001			
The role of the pharmaceutical companies in regard to PrEP is unclear	−0.25	(−0.44: −0.06)	0.011			
The use of PrEP will lead to a decrease in condom use and an increase in STI	−0.89	(−1.06: −0.71)	<0.001	−0.21	(−0.40: −0.02)	0.034
PrEP is cheaper than lifelong HIV treatment	0.42	(0.25: 0.59)	<0.001			
PrEP is an effective intervention to prevent HIV	0.88	(0.74: 1.02)	<0.001	0.45	(0.30: 0.61)	<0.001
PrEP prescription should be part of routine care at STI clinics	0.65	(0.52: 0.78)	<0.001	0.17	(0.04: 0.30)	0.013
There is not enough knowledge yet about PrEP	−0.60	(−0.81: −0.40)	<0.001			
Costs of PrEP are a problem	−1.01	(−1.18: −0.84)	<0.001	−0.28	(−0.51: −0.05)	0.019
I would worry that some people have to use PrEP lifelong	−0.41	(−0.55: −0.26)	<0.001			
It is unethical to prescribe ART to healthy individuals	−0.69	(−0.84: −0.55)	<0.001			
PrEP is a good addition to prevention strategies	1.13	(0.95: 1.31)	<0.001			
The costs of PrEP will not outweigh the number of HIV infection prevented	−0.33	(−0.52: −0.14)	0.001			
The STI clinic is not the right place for PrEP prescription	−0.71	(−0.93: −0.49)	<0.001			
Daily use of prevention strategies has already been tested	0.30	(0.13: 0.47)	0.001			

*ART, antiretroviral therapy; PrEP, pre-exposure prophylaxis; STI, sexually transmitted infection*.

In the final multivariable analyses among HIV specialists (Table 4), physicians were less likely to have a positive attitude toward PrEP implementation than nurses (β = −0.83, *p* = 0.008). Beliefs associated with a higher PrEP acceptability among all HIV specialists were “PrEP is an effective intervention to prevent HIV” (β = 0.49, *p* = 0.023) and “PrEP is a good addition to prevention strategies” (β = 0.28, *p* = 0.040). The belief associated with a lower PrEP acceptability was “it is unethical to prescribe ART to healthy individuals” (β = −0.31, *p* = 0.005).

**Table 4:** Determinants of acceptability of PrEP implementation in the Netherlands among 66 HIV specialists in the Netherlands (2015).

	Univariable analyses			Multivariable analyses		
	β	(95% CI)	*p*-value	β	(95% CI)	*p*-value
**Demographic characteristics**						
**Age**						
<40 years	Ref.		0.447			
40–49 years	−0.27	(−1.47: 0.93)				
50–59 years	0.10	(−1.17: 1.37)				
≥60 years	−0.97	(−2.48: 0.54)				

**Gender**						
Male	Ref.		0.819			
Female	−0.10	(−0.94: 0.75)				

**Profession**						
Nurse	Ref.		0.070	Ref.		0.008
Physician	−0.76	(−1.59: 0.06)		−0.83	(−1.44: −0.22)	

**Type of hospital**						
Academic	Ref.		0.603			
General	0.22	(−0.61: 1.05)				

**Length of employment within hospital**						
0–4 years	Ref.		0.503			
5–9 years	0.01	(−1.31: 1.34)				
10–14 years	0.02	(−1.27: 1.31)				
≥15 years	−0.72	(2.06: 0.62)				

**Number of HIV-positive patients in care**						
≤250	Ref.		0.589			
>250	0.23	(−0.62: 1.09)				

**Questions about PrEP from patients in the previous 6 months**						
No	Ref.		0.651			
Yes	0.12	(−0.40: 0.64)				

**Ever prescribed PrEP**						
No	Ref.		0.435			
Yes	0.61	(−0.94: 2.15)				

**Self-perceived knowledge of PrEP (7-point scale: 1 “very poor” to 7 “very good”)**						
Self-perceived knowledge of PrEP efficacy	0.26	(−0.12: 0.64)	0.175			
Self-perceived knowledge of PrEP side effects	0.05	(−0.26: 0.36)	0.742			
Self-perceived efficacy to identify target groups for PrEP	0.17	(−0.14: 0.48)	0.279			
Self-perceived capability of deciding to prescribe PrEP	0.12	(−0.18: 0.41)	0.431			

**General beliefs about PrEP (7-point scale: 1 “completely disagree” to 7 “completely agree”)**						
The use of PrEP will lead to a decrease in condom use and an increase in STI	−0.69	(−0.95: 0.43)	<0.001			
PrEP is an effective intervention to prevent HIV	1.09	(0.71: 1.47)	<0.001	0.49	(0.07: 0.92)	0.023
It is unethical to prescribe ART to healthy individuals	−0.54	(−0.75: −0.34)	<0.001	−0.31	(−0.53: −0.10)	0.005
PrEP is a good addition to prevention strategies	0.68	(0.47: 0.88)	<0.001	0.28	(0.01: 0.54)	0.040
I’m worried about the long-term side effects of PrEP	−0.18	(−0.46: 0.09)	0.187			
PrEP will lead to an increase in HIV resistance	−0.30	(−0.57: −0.03)	0.032			
Non-biomedical HIV interventions (i.e., behavioral) are better than PrEP	−0.23	(−0.52: 0.06)	0.125			
I’m worried about the short-term side effects of PrEP	0.04	(−0.24: 0.31)	0.789			

*ART, antiretroviral therapy; PrEP, pre-exposure prophylaxis; STI, sexually transmitted infection*.

### Daily versus Event-Driven PrEP Use

There was no preference for daily or event-driven PrEP, given the mean score of the combined beliefs about event-driven versus daily PrEP of 3.99 (SD 1.00) among STI professionals and 4.14 (SD 1.01) among HIV specialists (Table S2 in Supplementary Material).

### Key Populations, Serodiscordant Couples, and Reimbursement of PrEP Costs

**Figure 2:**
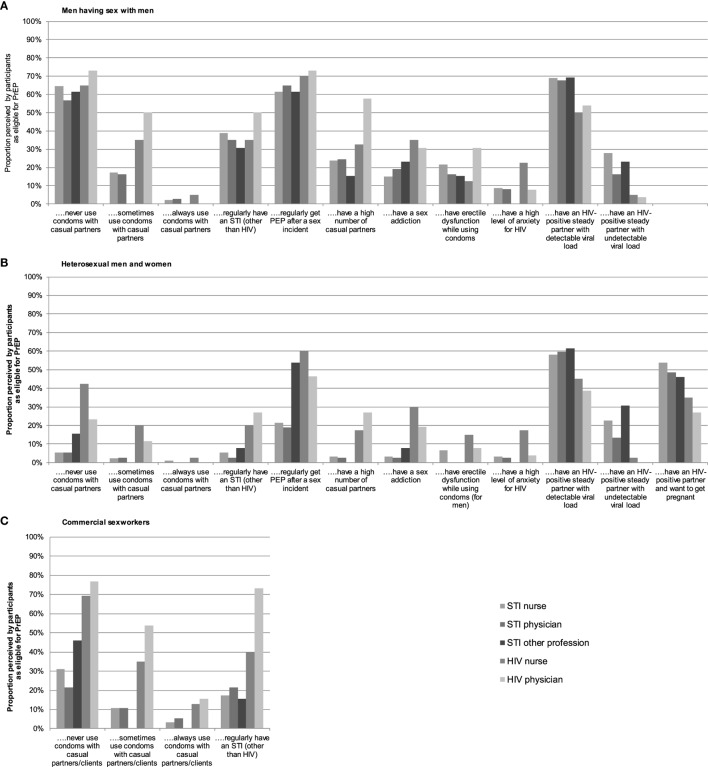
Beliefs among health-care professionals as to which key populations are eligible for pre-exposure prophylaxis (PrEP) in the Netherlands: **(A)** men having sex with men, **(B)** heterosexual men and women, and **(C)** commercial sex workers.

As shown in Figure 2, most participants believed the following groups to be eligible for PrEP: MSM who regularly get post-exposure prophylaxis after a sexual exposure (136/209, 65.1%), MSM who never use condoms with casual partners (134/209, 64.1%), and MSM or heterosexuals who have an HIV-positive steady partner with a detectable viral load (132/209, 63.2% and 112/209, 53.6%, respectively).

To prevent HIV transmission within serodiscordant couples, most HIV specialists preferred to treat only the HIV-positive partner (34/66, 51.5%) or to treat the HIV-positive partner while providing PrEP to the HIV-negative partner as long as the HIV-positive partner has a detectable viral load (26/66, 39.4%). A minority preferred only prescribing PrEP for the HIV-negative partner (2/66, 3.0%) or prescribing ART for the HIV-positive partner and PrEP for the HIV-negative partner without limited time frame (2/66, 3.0%), or just counseling (2/66, 3.0%). HIV physicians and nurses did not differ significantly in their preferences to prevent HIV transmission within serodiscordant couples.

As to PrEP costs, over half of STI professionals and HIV specialists (113/209, 54.1%) indicated that PrEP users should partially contribute, whereas 35.4% (74/209) indicated that PrEP users should pay all the costs; 5.7% (12/209) favored complete coverage by health insurance, 4.3% (9/209) were neutral, and 0.5% (1/209) indicated that the government should pay all the costs.

## Discussion

This cross-sectional study on PrEP acceptability and its determinants found only moderate PrEP acceptability among STI professionals and HIV specialists. These moderate levels of acceptability are worrisome as they may impede smooth implementation of PrEP in the Netherlands. Facilitating factors toward PrEP acceptability in the Netherlands were the beliefs that PrEP is an effective intervention to prevent HIV, PrEP prescription should be a part of routine care at STI clinics, PrEP is a good addition to prevention strategies, and that taking PrEP is better than getting HIV. Beliefs forming barriers to PrEP implementation were the perceived potential decrease in condom use and the increase in STI prevalence, the high costs of PrEP, and the belief that it is unethical to prescribe ART to healthy individuals. Previous studies on PrEP-related beliefs of health-care professionals in developed countries have identified concerns regarding drug resistance, low adherence, a rise in STI, long-term toxicity, and lack of country-specific guidance (11–15). Some of these findings are now corroborated by our data for the Dutch context.

In order to increase the acceptability of PrEP implementation among health-care professionals in the Netherlands, the beliefs that may form barriers for PrEP implementation need to be addressed and beliefs that could facilitate PrEP implementation need to be more broadly communicated. Regarding the concerns around the anticipated decrease in condom use and the increase in STI in PrEP users, open-label studies showed mixed results (16–21). If PrEP is implemented in the Netherlands, structured sexual-risk behavior counseling should be offered as the standard component of consultations (22). Addressing such concerns among health-care professionals and offering them tools to intervene on such topics among their clients may increase PrEP acceptability.

In our study and others across Europe (23), beliefs regarding the high costs of PrEP were likewise found to be a potential barrier for PrEP implementation. The costs of PrEP are indeed high and the implementation of PrEP therefore costly, but this barrier may be partly overcome if the study results that PrEP is most likely to be cost-effective and potentially even cost-saving are highlighted (10, 24, 25). In our study, over half of participants had the opinion that PrEP users need to contribute to the costs of PrEP. However, this was not further explored in relation to the amount that would need to be contributed and to the income. For those who cannot otherwise afford PrEP, the acceptability of public funding of costs was high in a study from the USA (26). The high costs have also shown to be a potential barrier for the use of PrEP by potentially eligible candidates in the Netherlands (27, 28). The problem of cost combined with the lack of a reimbursement system clearly requires a solution to enable successful PrEP implementation in the Netherlands and probably in other countries lacking PrEP reimbursement programs. The recent introduction of significantly cheaper generic PrEP, also in the Netherlands (29), could provide a partial answer to the growing demand from PrEP in countries that lack a reimbursement system.

Although adherence to PrEP and long-term side effects were important concerns for participants, they were in our analysis not associated with the acceptability of PrEP implementation in the Netherlands. This finding could reflect current professional knowledge that (1) even with suboptimal levels of adherence, PrEP still yields a high level of protection and does not lead to drug resistance (16); and (2) possible long-term side effects are likely to be non-severe (7, 30, 31).

Among STI professionals, self-perceived knowledge of PrEP efficacy and side effects was significantly lower compared to HIV specialists, perhaps a result of their different educational and professional background. An alternative explanation is that data were collected earlier among STI professionals than among HIV specialists, and the interim period saw new research outcomes of PrEP studies (5–7), publication of the world health organization PrEP guidelines (1), and publicity for the start of the Amsterdam PrEP project (32). These developments could explain differences in knowledge between the two professional groups. It is important to improve PrEP knowledge among STI professionals, particularly as future PrEP programs are likely to be implemented at STI clinics. Many MSMs at risk for HIV and STI make use of free STI testing and treatment at Dutch STI clinics (33), and therefore PrEP counseling and provision could easily be incorporated into this setting. Our results also show that among HIV specialists, the implementation of PrEP is more acceptable at STI clinics than at HIV treatment centers. Although HIV specialists have more knowledge regarding ART, people wanting PrEP are not normally in need of such expensive and specialized hospital-based knowledge or care.

In regard to the use of daily or event-driven PrEP, our study showed no clear preference among the respondents. This may reflect the timing of the survey, which was taken just after the early interruption of two large European PrEP studies (one among daily and one among event-driven PrEP users) that both showed 86% reduction in the number of new HIV infections (5, 6).

As for key eligible populations, health-care professionals focused on MSM at a high risk of sexual acquisition of HIV and heterosexuals with an HIV-positive partner with a detectable viral load. These findings align with other studies (34, 35) and with recommendations for PrEP use in guidelines (1–3), indicating that health-care professionals in STI clinics and HIV treatment centers can correctly identify individuals eligible for PrEP.

Strengths of this study are its nationwide character, its use of both nurses and medical doctors from two different settings, and its application of measurement tools that were empirically generated through a qualitative elicitation process. However, some limitations need to be addressed. First, the number of completed questionnaires and the response rate were relatively low, as is common in survey-based research (36). Nevertheless, people from almost all invited STI clinics responded, as did more than one-third of HIV specialists. Second, to increase the response rate in the latter group, they were provided with a shorter questionnaire than was given to STI professionals, which made the comparison between the two groups more limited. Third, we did not include general practitioners in our study. As it may be likely that PrEP is implemented at general practitioners, PrEP acceptability should also be explored among this group of health-care professionals.

In conclusion, health-care professionals in STI clinics and HIV treatment centers in the Netherlands have only a moderate level of acceptability toward PrEP implementation, and PrEP knowledge among STI professionals is suboptimal. This may impede the smooth implementation of PrEP in the Netherlands. The high costs of PrEP, worries about a possible decrease in condom use and an increase in STI, and the belief that it is unethical to prescribe ART to healthy individuals were identified as influential barriers for PrEP implementation and need to be addressed in order to successfully implement PrEP in the future. Furthermore, it is important to increase PrEP knowledge in educational programs for STI professionals.

## Ethics Statement

The questionnaire for health-care professionals was anonymous and did not request personal or sensitive data, and therefore approval by an ethics committee was not required, according to Dutch legislation.

## Author Contributions

Study concept and design: JB, EH, and UD. Data collection: JB, EH, FGDL, and UD. Statistical analysis: JB and UD. Drafting of this manuscript: JB and EH. Interpretation of the data and critical revision of the manuscript: all authors.

## Conflict of Interest Statement

The institute received financial reimbursement for the time served by EH on advisory boards of Gilead Sciences. For MP, the institute received independent research support and speaker’s fees from Gilead, AbbVie, MSD, and Roche. For UD, the institute received independent research support from Gilead, AbbVie, MSD, and ViiV. The Public Health Service of Amsterdam received PrEP medication from Gilead Sciences for the Amsterdam PrEP project and participated in a Gilead-sponsored study. All other authors declare that the research was conducted in the absence of any commercial or financial relationships that could be construed as a potential conflict of interest.
