# Supplementary material for: The Acceptability of Pre-Exposure Prophylaxis: Beliefs of Health-Care Professionals Working in Sexually Transmitted Infections Clinics and HIV Treatment Centers
Source: Front Public Health. 2018 Feb 9;6:5. doi: 10.3389/fpubh.2018.00005 (PMC5811525; doi:10.3389/fpubh.2018.00005)
Supplement: Supplementary file 1 [file Data_Sheet_1.PDF]

Supplementary Material

Bil JP, Hoornenborg E, Prins M, Hogewoning A, Dias Goncalves Lima F, de Vries HJC and Davidovich U (2018) The Acceptability of Pre-Exposure Prophylaxis: Beliefs of Health-Care Professionals Working in Sexually Transmitted Infections Clinics and HIV Treatment Centers. Front. Public Health 6:5.doi: 10.3389/fpubh.2018.00005

TABLE S1 | Overview of PrEP beliefs, measured items and reliability of items measured among STI professionals and HIV specialists, the Netherlands (2015)

Beliefs and items measured among STI professionals		
**Beliefs**	**Items**	**Correlation**[1]
It is unclear who has to pay for PrEP	I belief it is to unclear who should pay for PrEP	NA
Taking PrEP is better than getting HIV	I belief that taking PrEP is better than getting HIV	NA
Adherence to PrEP will be insufficient	I would worry about adherence to PrEP when providing PrEP	0.72
	I would worry about the misuse of PrEP	
The role of the pharmaceutical companies in regards to PrEP is unclear	I would worry about the role of the pharmaceutical companies when providing PrEP	NA
The use of PrEP will lead to a decrease in condom use and increase in STIs	I belief that providing PrEP stimulates risk behavior	0.90
	I belief that providing PrEP decreases personal responsibility to prevent HIV	
	I belief the use of PrEP will lead to a decrease in condom use	
	I belief PrEP is a carte blanche to stop using condoms	
	I belief PrEP will change the norm in regards to condom use	
	I belief that is more difficult to use condoms after someone has stopped using PrEP	
	I belief that the use of PrEP will lead to an increase in the number of STIs	
	As there will be an increase in STIs due to PrEP, I belief that there is an increased HIV transmission risk instead of decreased.	
PrEP is cheaper than life long HIV treatment	I belief PrEP using PrEP is cheaper than using life long HIV treatment	NA
PrEP is an effective intervention to prevent HIV	I belief that PrEP is an effective intervention for individuals to prevent HIV	0.67
	I belief PrEP is an effective intervention to reduce the number of new HIV infections in the Netherlands	
PrEP prescription should be part of routine care of STI clinics	I belief it is important that the STI clinic supports new developments, such as PrEP	0.82
	I belief that PrEP prescription should be part of routine care of STI clinics	
There is not enough knowledge yet about PrEP	I belief that there is not enough knowledge yet about the long term effects of PrEP to start providing PrEP at the STI clinic	0.82
	I belief that they key populations for PrEP is to unclear yet	

	I would worry about the efficacy of PrEP	
	I would worry about the long term side effects of PrEP when providing PrEP	
	I would worry about ART resistance when providing PrEP	
Costs of PrEP are a problem	I belief there is not enough public support to provide PrEP	0.80
	I belief there is not enough public support for the coverage of PrEP costs	
	I belief it is better to spent money and resources on other interventions than PrEP	
	I belief the costs of treating other STIs will increase due to the use of PrEP	
	I belief that you should not provide expensive drugs to individuals that are not willing to have safe sex	
I would worry that some people have to use PrEP life long	I would worry that some individuals would use PrEP life long when providing PrEP	NA
It is unethical to prescribe antiretroviral therapy to healthy individuals	I belief it is unethical to prescribe healthy individuals daily pills to prevent HIV	0.91
	I belief that by providing PrEP we increase medicalization	
	I belief taking medication to support sexual choices is unwanted	
	I belief taking PrEP lifelong by healthy individuals is worrisome	
	I belief taking PrEP temporarily by healthy individuals is worrisome	
PrEP is a good addition to prevention strategies	I belief PrEP is a good addition for individuals that are not able to use condoms consistently	0.81
	I belief PrEP is a good addition for individuals in a serodiscordant relationship	
	I belief PrEP is a good addition for individuals with high HIV anxiety	
	I belief PrEP is a good addition to increase the quality of sex life	
	I belief PrEP is unnecessary as there are better alternatives to prevent HIV [2]	
	I belief that providing PrEP will interfere with current counseling strategies [2]	
	I belief that PrEP has the advantage to provide extra counseling to those at high risk of HIV	
	I belief that individuals who are not able to use condoms consistently are also not able to consistently use daily drugs [2]	
	We should provide both daily and event-driven PrEP as I belief that this increases the choice of HIV prevention options	
The costs of PrEP will not outweigh the number of HIV infections prevented	I belief the costs of PrEP will not outweigh the number of HIV infections prevented	NA
The STI-clinic is not the right place for PrEP prescription	I belief the STI clinic has the moral obligation to do research on PrEP [2]	0.72
	I belief providing PrEP complicates current guidelines	
	I belief the STI clinic lacks capacity to prescribe PrEP	
	I belief the STI clinic lacks the knowledge and ability to provide counseling for PrEP adherence	

	I belief the STI clinic is the right place to reach the key populations for PrEP [2]	
Daily use of prevention strategies has already been tested before	I would not worry about adherence because daily use of drugs for the prevention of conditions has already been proven to work	NA

Beliefs and items measured among HIV specialists

Beliefs	Items [3]	Correlation[1]
The use of PrEP will lead to a decrease in condom use and increase in STIs	I belief the use of PrEP will lead to a decrease in condom use	0.83
	I belief that the use of PrEP will lead to an increase in the number of STIs	
PrEP is an effective intervention to prevent HIV	I belief that PrEP is an effective intervention for individuals to prevent HIV	0.73
	I belief PrEP is an effective intervention to reduce the number of new HIV infections in the Netherlands	
	As there will be an increase in STIs due to PrEP, I belief that there is an increased HIV transmission risk instead of decreased [2]	
	I belief PrEP will be cost effective.	
	I'm belief that there is not enough knowledge yet about PrEP in a real-life setting [2]	
	I'm worried that PrEP is not 100% effective [2]	
	I'm worried about PrEP adherence [2]	
It is unethical to prescribe antiretroviral therapy to healthy individuals (trans)	I belief it is unethical to prescribe healthy individuals daily pills to prevent HIV	NA
PrEP is a good addition to prevention strategies	I belief PrEP is unnecessary as there are better alternative to prevent HIV [2]	NA
I'm worried about the long term side effects of PrEP	I'm worried about the long term side effects of PrEP	NA
PrEP will lead to an increase in HIV resistance	I belief that the use of ART for prevention will lead to an increase in HIV resistance	NA
Non-biomedical HIV interventions (i.e. behavioral interventions) are better	I think non-biomedical HIV intervention like behavioral intervention are better than PrEP	NA
I'm worried about the short term side effects of PrEP	I'm worried about the short term side effects of PrEP	NA

All items and beliefs were measured on a 7-point scale ranging from 1"Completely disagree" to 7"Completely agree".
The questionnaire among STI clinic professionals included all PrEP beliefs measured in the focus group discussions. The questionnaire among HIV treating specialists only included major themes that were derived from the analyses from the focus group discussions.
[1] Items were combined if they measured the same belief and had acceptable correlation (2 items: Spearman rho ≥0.6 and $p<0.05$; >2 items: Cronbach's alpha ≥0.7)
[2] The reversed scale was used to combine this item with the other items in the associated belief
PrEP: pre-exposure prophylaxis, STI: sexually transmitted infection

TABLE S2 | Beliefs about event-driven versus daily PrEP among 209 health care providers, the Netherlands (2015).

	STI professionals									HIV specialists							STI professionals versus HIV specialists
	Total (N=143)		Nurse (n=93)		Physician (n=37)		Other (n=13)		STI nurse versus STI physician vs. STI other	Total (N=66)		Nurse (n=40)		Physician (n=26)		HIV nurse versus HIV physician	
**Items**	**Mean**	**SD**	**Mean**	**SD**	**Mean**	**SD**	**Mean**	**SD**		**Mean**	**SD**	**Mean**	**SD**	**Mean**	**SD**		
Event-driven PrEP is a better option than daily PrEP	4.14	1.48	4.08	1.56	4.35	1.42	4.00	1.00	$F(2,140)=0.52$, $p=0.593$	4.26	1.43	4.23	1.19	4.31	1.76	$t(64)=0.23$, $p=0.820$	$t(207)=-0.54$, $p=0.589$
Event-driven PrEP is easier to maintain than daily PrEP	4.38	1.47	4.35	1.49	4.54	1.48	4.15	1.34	$F(2,140)=0.38$, $p=0.683$	4.77	1.61	4.83	1.48	4.69	1.81	$t(207)=-0.33$, $p=0.746$	$t(207)=-1.72$, $p=0.087$
Daily PrEP is more effective than event-driven PrEP[1]	4.39	1.27	4.24	1.14	4.78	1.47	4.38	1.45	$F(2,140)=2.50$, $p=0.086$	4.18	1.49	4.50	1.32	3.69	1.62	$t(207)=-2.22$, $p=0.030$	$t(207)=1.05$, $p=0.295$
Event-driven PrEP will be preferred over daily PrEP because it will be cheaper	4.52	1.31	4.43	1.38	4.54	1.17	5.08	1.11	$F(2,140)=1.41$, $p=0.249$	4.41	1.60	4.35	1.56	4.50	1.68	$t(207)=0.37$, $p=0.713$	$t(207)=0.52$, $p=0.605$
Event-driven PrEP will be less of a burden than daily PrEP	4.59	1.42	4.48	1.38	4.92	1.38	4.38	1.71	$F(2,140)=1.40$, $p=0.249$	4.73	1.33	4.78	1.35	4.65	1.32	$t(207)=-0.36$, $p=0.721$	$t(207)=-0.68$, $p=0.497$
I have less objections against providing event-driven PrEP than against providing daily PrEP	3.55	1.72	3.53	1.69	3.57	1.80	3.62	1.80	$F(2,140)=0.02$, $p=0.981$	3.52	1.82	3.75	1.58	3.15	2.11	$t(207)=-1.31$, $p=0.195$	$t(207)=0.12$, $p=0.908$
Event-driven PrEP is more complicated to use correctly than daily PrEP1	4.87	1.45	4.84	1.42	4.97	1.48	4.85	1.72	$F(2,140)=0.11$, $p=0.892$	4.50	1.63	4.73	1.52	4.15	1.76	$t(207)=-1.40$, $p=0.166$	$t(207)=1.66$, $p=0.098$
Overall: event-driven PrEP is preferred over daily PrEP[2]	3.99	1.00	3.97	1.03	4.02	0.95	4.00	0.99	$F(2,140)=0.04$, $p=0.964$	4.14	1.01	4.10	0.91	4.21	1.17	$t(64)=-0.42$, $p=0.673$	$t(207)=-1.05$, $p=0.297$

All items and beliefs were measured on a 7-point scale ranging from 1"Completely disagree" to 7"Completely agree".
[1] The reversed scale was used to combine this item with the other items in the overall belief "event-driven PrEP is preferred over daily PrEP"
[2] Includes all items measured among STI clinic professionals and HIV treating specialists (STI clinic professionals: Cronbach's alpha=0.81; HIV treating specialists: Cronbach's alpha=0.77).
PrEP: pre-exposure prophylaxis, STI: sexually transmitted infection, SD: standard deviation
